# Effect of sludge age on methanogenic and glycogen accumulating organisms in an aerobic granular sludge process fed with methanol and acetate

**DOI:** 10.1111/1751-7915.12292

**Published:** 2015-06-08

**Authors:** M Pronk, B Abbas, R Kleerebezem, M C M van Loosdrecht

**Affiliations:** Department of Biotechnology, Delft University of TechnologyDelft, The Netherlands

## Abstract

The influence of sludge age on granular sludge formation and microbial population dynamics in a methanol- and acetate-fed aerobic granular sludge system operated at 35°C was investigated. During anaerobic feeding of the reactor, methanol was initially converted to methane by methylotrophic methanogens. These methanogens were able to withstand the relatively long aeration periods. Lowering the anaerobic solid retention time (SRT) from 17 to 8 days enabled selective removal of the methanogens and prevented unwanted methane formation. In absence of methanogens, methanol was converted aerobically, while granule formation remained stable. At high SRT values (51 days), *γ-Proteobacteria* were responsible for acetate removal through anaerobic uptake and subsequent aerobic growth on storage polymers formed [so called metabolism of glycogen-accumulating organisms (GAO)]. When lowering the SRT (24 days), *Defluviicoccus*-related organisms (cluster II) belonging to the *α-Proteobacteria* outcompeted acetate consuming *γ-Proteobacteria* at 35°C. DNA from the *Defluviicoccus*-related organisms in cluster II was not extracted by the standard DNA extraction method but with liquid nitrogen, which showed to be more effective. Remarkably, the two GAO types of organisms grew separately in two clearly different types of granules. This work further highlights the potential of aerobic granular sludge systems to effectively influence the microbial communities through sludge age control in order to optimize the wastewater treatment processes.

## Introduction

Aerobic granular sludge (AGS) is a new emerging wastewater treatment technology that provides several advantages over conventional activated sludge-based systems, such as reduction of surface area, lower energy demand, lower reactor volume, easier process control and excellent effluent quality (Morgenroth *et al*., 1997; Wilderer and McSwain, 2004; de Kreuk and van Loosdrecht, 2006). A short settling time imposes a selective pressure for compact dense granules at the expense of flocculent sludge. Aerobic granules allow for simultaneous nitrogen, chemical oxygen demand (COD) and phosphate removal in one reactor compartment, excluding the need for recirculation loops resulting in a significant reduction of energy consumption (De Kreuk *et al*., 2007; Isanta *et al*., 2012; Lochmatter and Holliger, 2014). AGS systems depend on the anaerobic uptake of carbon sources and the subsequent storage of polyhydroxyalkanoates (PHA) to prevent growth of fast growing strict aerobic heterotrophic bacteria (De Kreuk and van Loosdrecht, 2004; Weissbrodt *et al*., 2013a). This is a key strategy to achieve long-term stable granulation and stable nitrogen and phosphorus removal.

Pronk and colleagues (2015) showed that the operational conditions in AGS systems fed with methanol might provide an opportunity for methanogens to proliferate. This is mostly because the formation of storage polymers from methanol is challenging under the conditions that are common in AGS systems (Dobroth *et al*., 2011). Methanol is present in many industrial wastewaters, since it is a key solvent in many processes. Treatment of such wastewaters could consequently lead to unwanted formation of methane, a greenhouse gas and potentially explosive when mixed with air. Controlling the anaerobic solid retention time (SRT) was suggested as a potential effective method to prevent proliferation of methanogens feeding on methanol or other single carbon compounds that do not lead to storage polymer formation and are excellent precursors for biogas production (Pronk *et al*., 2015).

Acetate has been the main carbon source in AGS reactors operated in laboratories (De Kreuk *et al*., 2005; Carucci *et al*., 2008; Weissbrodt *et al*., 2013b). Its influence on the microbial population has been well described. In most experiments, it leads to the proliferation of polyphosphate-accumulating organisms (PAO) or glycogen-accumulating organisms (GAO). This is mostly due to anaerobic/aerobic regimes, temperature, SRT, phosphate-limiting conditions, etc. (Lopez-Vazquez *et al*., 2009b; Barr *et al*., 2010; Winkler *et al*., 2011; Rocktäschel *et al*., 2013). However, temperature experiments in AGS have generally focused on the competition between PAO and GAO as this is of vital importance for phosphate removal during sewage treatment (De Kreuk *et al*., 2005; Bassin *et al*., 2012). Very few reports focus on the competition between different types of GAOs in AGS reactors especially at temperatures above 30°C, as is common in industrial wastewaters. Furthermore, previous studies did not unambiguously clarify the effect of elevated temperatures (30°C and 35°C) on the stability of AGS. Ebrahimi and colleagues (2010) report poor granulation and incomplete removal of acetate during the anaerobic feeding while Pronk and colleagues (2015) reports excellent granulation and full acetate consumption during the anaerobic feeding period at 35°C. Cui and colleagues (2014) also report stable granulation, albeit with an aerobic feeding regime. Until now, the fate of acetate fed anaerobically in the AGS process at mesophilic temperatures has not been clarified yet.

In this study, we investigated the conversion of methanol and acetate by AGS at 35°C, with an emphasis on preventing methanogenic conversion. We hypothesized that methanogens are effectively washed out from the system by lowering the anaerobic SRT. Hereto, the average SRT in an AGS bioreactor was reduced from originally 51 days to 24 days, resulting in an anaerobic SRT of approximately 8 days. Additionally, the effect of the decreasing SRT on the acetate-consuming (GAO) population was intensively monitored.

## Results

### The influence of SRT on methylotrophic methanogens

**Figure 1:**
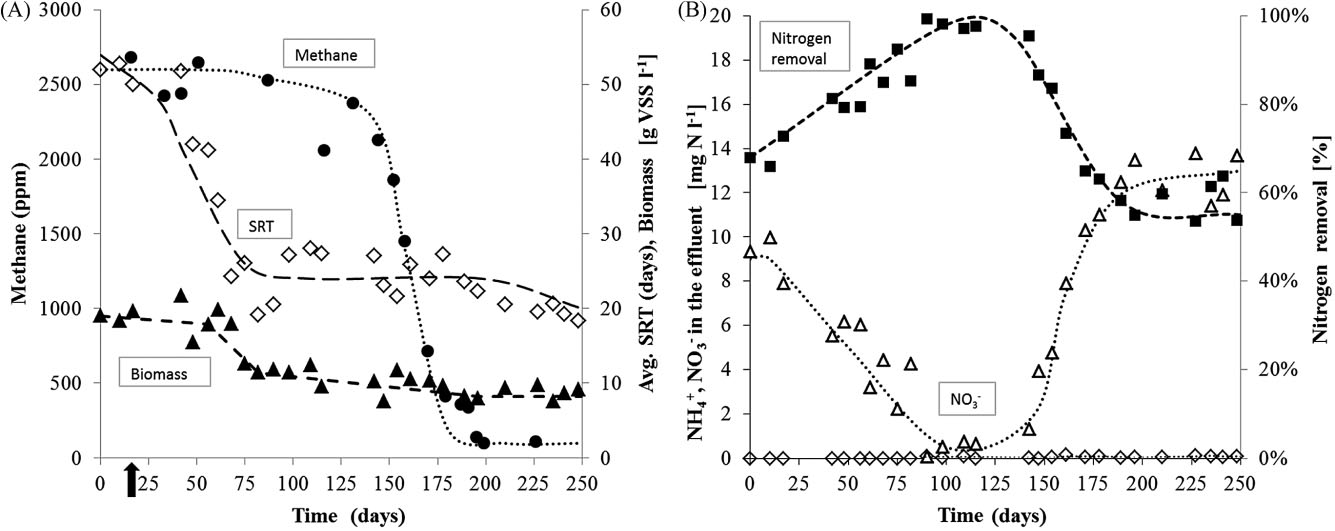
(A) Evolution in time of methane concentration measured just after the anaerobic feeding period when the aeration started (filled circle), average anaerobic solid retention time (days) (open diamond) and biomass concentration (g VSS l^−1^) (filled triangle) in an aerobic granular sludge reactor fed with acetate and methanol at 35°C. (B) Nitrogen species in the effluent: ammonium in the effluent (open diamonds), nitrate in the effluent (open triangles), nitrogen removal efficiency (closed squares). Arrow indicates when the solid retention time of 24 days was introduced. Lines are shown to indicate trends.

The reactor was inoculated with granular sludge from a previous experiment in which the influent COD consisted of a mixture of organic substrates including 15% of methanol. During these previous experiments, methanol was fully utilized during the anaerobic feeding period by methanogens, resulting in high methane emissions in the off-gas due to stripping at the start of the aeration period. The responsible methanogenic organism was identified by Denaturing Gradient Gel Electrophoresis (DGGE) as *Methanomethylovorans uponensis* (Pronk *et al*., 2015). To evaluate this methanol conversion in more detail and to investigate the conditions inhibiting methanogenesis in AGS systems, the medium was simplified to a mixture of acetate (85 COD %) and methanol (15 COD %). By increasing the acetate concentration, the volumetric COD loading was kept constant (760 mg COD l^−1^ day^−1^) (Table 1). Acetate was fully consumed during the anaerobic feeding period over the course of the experiment. No degradable soluble COD was detected once the aeration period started. Also in these conditions, methanol was entirely converted to methane by methanogens during the anaerobic feeding period. Online gas analysis revealed methane concentrations of approximately 2600 ppm being released right after aeration was started (Fig. 1A). The COD-equivalent amount of methane produced was similar to the amount of methanol converted. After 17 days of operation, weekly removal of biomass from the reactor at the end of the aeration period was introduced in order to obtain an average total SRT of 24 days. This decreased the anaerobic SRT from approximately 17 to 8 days (Fig. 1A). Initially, methane emissions remained high due to the overcapacity for methanogenesis in the system and slow removal of methanogens from the reactor through SRT control. Only after 150 days of operation at reduced SRT, a decline in methane production was observed. The methanol concentration in the bulk liquid after the feeding increased simultaneously. This was accompanied by a higher oxygen consumption of the system. This indicates that the methanol was not used anaerobically anymore, but was instead converted aerobically. Aerobic conversion of methanol, however, did not lead to any significant visual granule deterioration. The average granule size did decrease after introduction of the shorter SRT regime from 1.7 mm (day 17) to 0.6 mm (day 226), but this had no adverse effect on the COD effluent quality. The system was fully nitrifying to nitrate and partial simultaneous denitrification (not optimized) occurred (Fig. 1B). Nitrogen removal efficiency increased from 65% at the start of the experiment to about 99% on day 90. This was most probably induced by a lower biomass concentration in the reactor and thus an increase in storage polymer formation per biomass, which then became available for denitrification. Methanol that was not converted anaerobically could also contribute to the denitrification either as a carbon source or by creating a larger anoxic zone in the biofilm due to the consumption of oxygen at the granule surface (see Figs S1 and S2). However, since methanol was quickly consumed during the first 5–10 min of the aerobic period, it is unlikely to have contributed significantly to the denitrification. The nitrogen removal efficiency gradually decreased from 98% on day 135 to 54% on day 190. A smaller granule size coupled with an increase of oxygen penetration depth in the granules leading to less denitrification potential was the most likely reason for this decline.

**Table 1:** Medium composition used in the cultivation of granular sludge

Medium composition	
Carbon medium (A)	(mM)
Acetate	67.6
Methanol	15.6
Nitrogen medium (B)	(mM)
NH_4_Cl	21.1
K_2_HPO_4_	0.7
KH_2_PO_4_	0.4
MgSO_4_._7_H_2_O	3.6
KCl	4.7
–	(ml l^−1^)
Trace element solution	9

### Segregation of two types of granules

**Figure 2:**
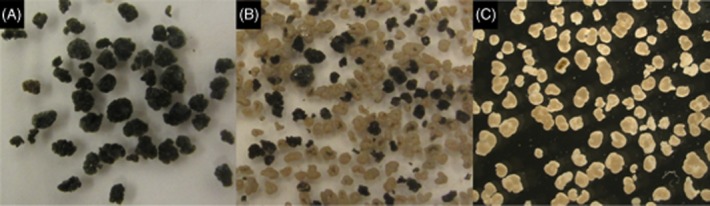
(A) black granules at the start of the experiment, (B) black and white granules after 120 days of operation at reduced SRT, and (C) white granules after 250 days with a solid retention time of 24 days fed with acetate (85% COD) and methanol (15% COD). Average granule size distribution for A, B and C were 1.7, 0.9 and 0.6 mm respectively.

A remarkable phenomenon was observed after lowering the total SRT from 51 to 24 days (17 and 8 days anaerobic respectively): white smaller granules started to develop separately from the already present large black granules. In the beginning of the experiment, separate white granules were rarely observed, and they were mostly attached as small white colonies to the outside of the large black granules. Over the course of this experiment, the white granules slowly started to dominate the reactor (Fig. 2).

**Figure 3:**
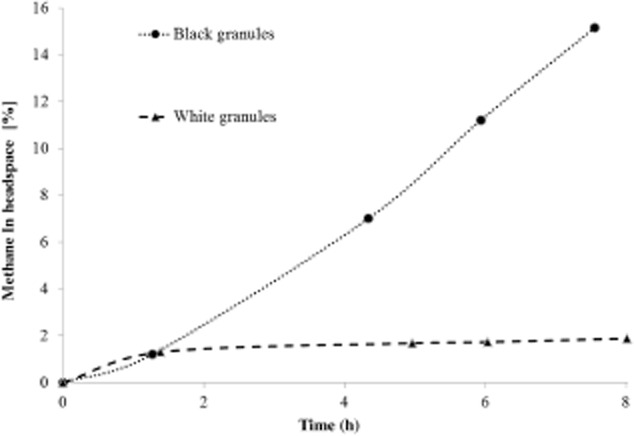
Methane measured in the headspace during anaerobic batch tests with methanol on selected white and black granules taken at day 140.

Besides the obvious difference in colour, also the morphology of the white granules differed strongly from the morphology of the black ones. White granules had a more open structure than the black granules, even though the settling rate was similar, as indicated by the black and white mixed granule bed after settling. In order to elucidate the difference between the two types of granules, anaerobic batch tests were performed with manually separated black and white granules. Under anaerobic conditions, 20 mM methanol was dosed to investigate methanogenic activity in both types of granules. Although the separation of black and white granules was based on colour only, a distinctive difference in conversion was observed. The black granules were responsible for the anaerobic conversion of methanol to methane, while the white granules had no methanogenic activity at all (Fig. 3). The maximum methane production rate in the black granules was 19.2 mmol CH_4_ (g VSS d^−1^).

### Microbial community analysis

In the black granules, *M. uponensis* was previously detected as the dominant methanogen responsible for the anaerobic conversion of methanol to methane (Pronk *et al*., 2015). From the white granules, no archaeal polymerase chain reaction (PCR) products were retrieved. Moreover, fluorescence of co-enzyme F_420_ present in *M. uponensis* was only observed during microscopy in the black granules. Adjacent to the apparent difference in methanogenic activity and colour, also the microbial community structure was investigated separately in black and white granules.

**Figure 4:**
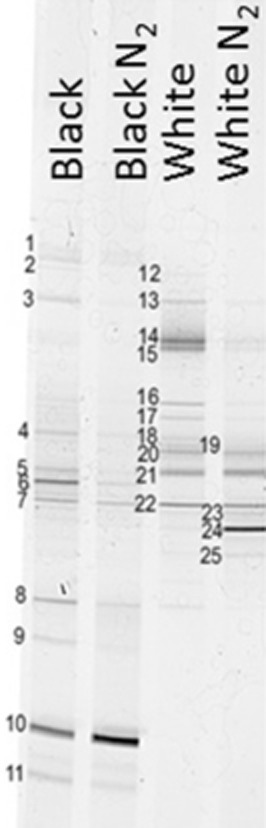
DGGE on bacterial 16s rDNA gene isolated from black and white granules (day 120) from an aerobic granular sludge reactor extracted with the protocol described in the standard extraction kit from (Mobio, USA) and the adjusted liquid nitrogen (N_2_) extraction method (as described in *Experimental procedures*).

**Figure 5:**
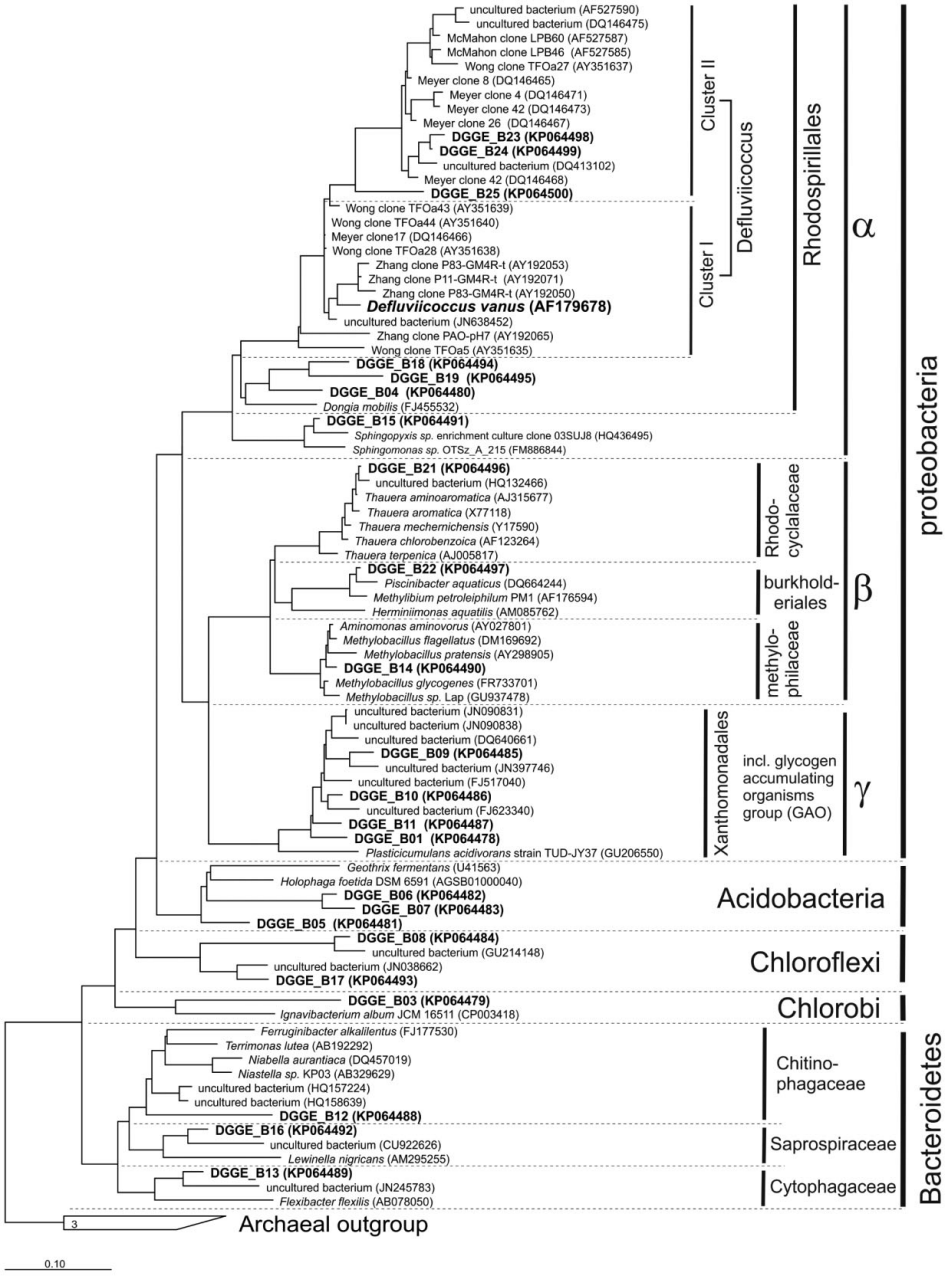
The phylogenetic tree was calculated using maximum-likelihood algorithm implemented in ARB (RAxML module). Full sequences from the SSU115-NR99 database were used for calculation together with an SSUref: bacteria filter (resulting in 1125 base pairs). DGGE bands and clones with a variable length (311–962 base pairs) were added later using parsimony algorithm. Bootstrap (250rxn) was performed but not shown. Sequences shown were deposited into GenBank under Accession No. KP064478–KP064500.

PCR-DGGE analysis was performed based on the 16S rRNA gene of bacteria to determine the microbial community structure. Figure 4 shows the banding pattern obtained for manually separated black and white granules taken at day 150. The profile shows a limited number of bands at the same height in both granules suggesting a significant difference in community structure. The sequenced bands are numbered B1 to B25, in both the banding pattern (Fig. 4) and the phylogenetic tree (Fig. 5).

### *α-Proteobacteria* and *γ-Proteobacteria*

**Figure 6:**
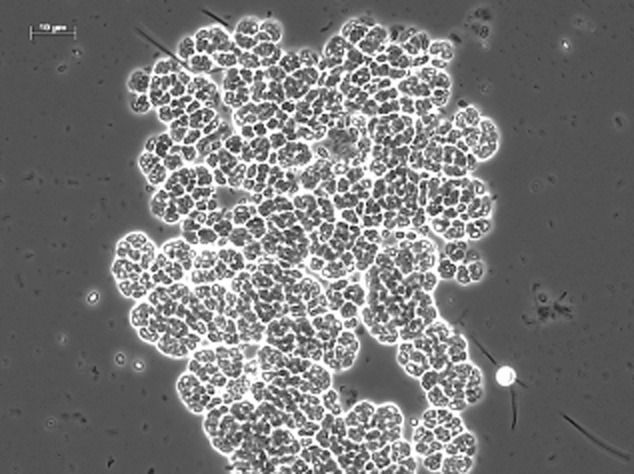
Microscopic examination of crushed white granules showing the tetrad-forming *Defluviicoccus*-related organisms in phase contrast.

Black granules were populated by *γ-Proteobacteria* (B1, B9, B10 and B11). Remarkably, the white granules seemed devoid of *γ-Proteobacteria*. Instead, *β-Proteobacteria* of the genus *Thauera* colonized the white granules (B21). However, Fluorescence In-Situ Hybridization (FISH) staining with a beta-probe failed to verify a significant presence of *Thauera* sp.-related species in the white granules, suggesting that part of acetate-consuming microorganisms were not identified. Furthermore, microscopic examination of the white granules unmistakably revealed tetrad-forming bacteria (Fig. 6). It was then assumed that a bias had occurred during extraction of the DNA. Therefore, the standard DNA extraction protocol was changed by including (among others) a liquid nitrogen step (see *Experimental procedures* for details). With this modified extraction protocol, a new strong band (B24) appeared in the DGGE gel. Once sequenced, it was identified as a member of the *Defluviicoccus* cluster II in the *α-Proteobacteria* and is a known tetrad-forming organism (TFO). The very weak band B23 is closely related (98.6%) to band B24. Bands B24 and B25 only have 94.5% similarity, nevertheless bands B23, B24 and B25 all belong to the genus *Defluviicoccus* sp. in cluster II. The closest cultivated species to B24 is *Defluviicoccus vanus*, but it has only 92% 16S-rRNA gene similarity and belongs to in cluster I. *Defluviicoccus* was not found in the black granules where *γ-Proteobacteria* were dominating (Fig. 4). Bands B14, B15 and B22 were also exclusively present in the white granules. Band B14 is most closely affiliated with *Aminomonas aminovorus* (99.1%) in the *Methylophilaceae* family, which are known for their methylotrophic capabilities. Methylotrophic potential is also present within the family *Burkholderiales* (B22). Band B15 belongs to the genus *Sphingopyxis*.

### Fluorescent *in situ* hybridization

**Figure 7:**
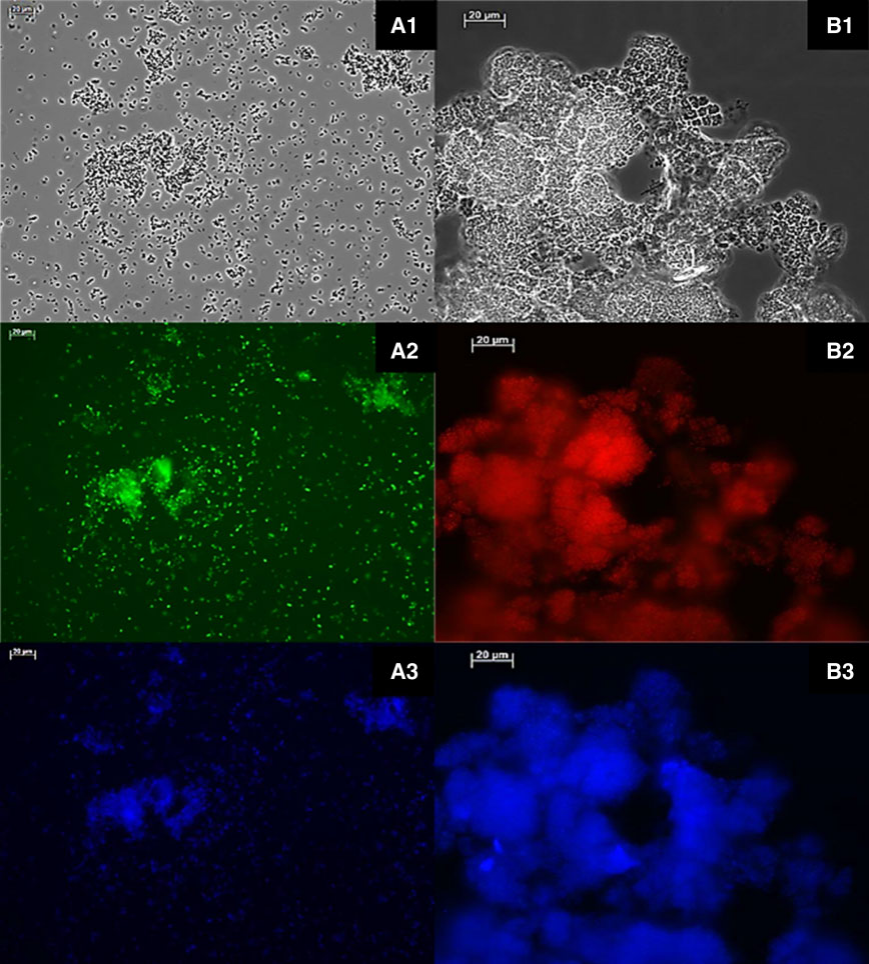
FISH performed on a black granule with (A1) phase-contrast, (A2) GAOmix targeting *γ-Proteobacteria* (fluos), (A3) EUBmix targeting all bacteria (Cy5); white granule (B1) phase contrast, (B2) *DF* 2 targeting *α-Proteobacteria* (Cy3), (B3) EUBmix targeting all bacteria (Cy5).

To gain more insight into the bacterial abundance and diversity in both types of granules, FISH staining was performed. In the black granules, in agreement with the DGGE results, the *γ-Proteobacteria* were visibly dominant (Fig. 7A). In the white granules, however, hardly any (< 5%) *γ-Proteobacteria* could be detected. Probes DF1 and DF2 targeting tetrad-forming *α-Proteobacteria* of *Defluviicoccus* clusters I and II, respectively, were used. Only probe DF2 successfully stained the bacteria in the different samples, confirming the DGGE results. In the white granules, the tetrad-forming *Defluviicoccus-*related bacteria were a dominant form (Fig. 7B).

## Discussion

### Preventing growth of methanogens

Methanogenesis from e.g. methanol during anaerobic feeding is an unwanted process during AGS treatment of industrial wastewater. When aeration starts, a large fraction of methane, a potent greenhouse gas, will be stripped to the air. Moreover, methane accumulation might also lead to safety risks and poor plug flow conditions during the anaerobic feeding. Control of the methanogenic archaea is therefore essential for effective implementation of the AGS process.

Most methanogens are very sensitive towards high redox, but this sensitivity varies widely depending on the species (Kiener and Leisinger, 1983; Morozova and Wagner, 2007). Methanogens are relatively slow-growing archaea (0.03–0.06 h^−1^ for *M. uponensis* on methanol) and as suggested previously by Pronk and colleagues (2015), lowering the anaerobic SRT below 10 days indeed resulted in the successful washout of *M. uponensis* from the AGS system. The relative long washout time needed to remove the methylotrophic methanogens was mainly due to a large population initially present combined with an SRT of 24 days. The reason might be that the SRT calculated is an average SRT. Biomass in the effluent contributes significantly to the biomass wasted from the system. Effluent biomass concentrations might seem insignificant (0.04–0.1 g l^−1^), but with 12 l of effluent per day, this accounts for nearly 20–30% of the waste sludge. This has a large impact on the SRT, lowering it significantly. Therefore, the SRT calculated here (24 days) is derived from the biomass that is removed deliberately as well as by effluent extraction. The SRT of bacteria present in granules might be higher than the SRT calculated for the entire system since the effluent mainly contains suspended or flocculent material. Because of this, washout times can be prolonged significantly compared with suspended systems.

The growth rate of *Methanomethylovorans* members on methanol was reported by to be in the range of 0.03–0.06 h^−1^ (Lomans *et al*., 1999; Cha *et al*., 2013). This should be more than sufficient to remain in the reactor, even when running at the lowest anaerobic SRT (8 days). However, this growth rate applies in optimal conditions only. In this experiment, the conditions are not optimal for methanogenic growth. Long aeration times would most definitely lead to oxygen inhibition and result in decay of the methanogenic archaea. The washout of the methanogens was most probably also further facilitated by the decreased granule size, since smaller granules allow for a relative deeper oxygen penetration depth (Kishida *et al*., 2006; Vázquez-Padín *et al*., 2010). Because of this strategy, methanol was not consumed during the anaerobic feeding period, but it was oxidized in the aerobic period. Usually, aerobic growth on readily available COD will result in fast heterotrophic growth and instable granulation (De Kreuk and van Loosdrecht, 2004; Kishida *et al*., 2006). However, aerobic growth on methanol is relatively slow and has also led to compact biofilms in other research (Villaseñor *et al*., 2000). This is in line with the general hypothesis on biofilm structure formation that suggests that low growth rates result in dense and compact biofilms (Van Loosdrecht *et al*., 1995; 1997).

In order to prevent methylotrophic methanogens in aerobic granular systems, the anaerobic SRT should be kept low enough, preferable below 10 days with wastewaters of 30°C to 40°C. Likely, under full-scale conditions, this is the standard operational condition. Oxygen concentrations during the aerobic period can also be increased to minimize growth of methanogens. Special care has to be taken to control the SRT in regard to segregation as a result of granule size differences if present (Winkler *et al*., 2011). As bigger granules provide more protection against oxygen for the methanogens, their proliferation might be more effectively prevented by removing bigger granules from the bottom of the reactor first.

### Competition between α- and γ*-*Proteobacterial GAOs for acetate

Acetate was fully converted to storage polymers during the anaerobic feeding period. Aerobic and anoxic growth occurred subsequently during the aerated mixing period. This anaerobic formation of PHA from volatile fatty acids and the subsequent growth once oxygen is available suggests that microorganisms performing the so-called GAO metabolism were present in the reactor. DGGE and FISH analysis identified *Defluviicoccus* cluster II members of the *α-Proteobacteria* as the major bacterial population. Most of the *Defluviicoccus-*related organisms found until now are reported to perform the GAO metabolism on acetate, propionate and lactate (Wong and Liu, 2007). They have been suggested as effective competitors for PAOs in the activated sludge process (Burow *et al*., 2007; Lanham *et al*., 2008; Mielczarek *et al*., 2013).

Tetrad-forming bacteria in the α-GAOs of genus *Defluviicoccus*, a novel cluster within its subgroup II, outcompeted the γ-GAOs for anaerobic acetate uptake when the SRT was lowered. Although the γ-GAOs and *Defluviicoccus*-related organisms have a highly similar metabolism, it has been reported that *Defluviicoccus*-related organisms are mainly proliferating when propionate is the main carbon source (Oehmen *et al*., 2005; Meyer *et al*., 2006). Acetate uptake rates of *Defluviicoccus*-related organisms have been reported as being much lower than for the γ-GAOs (Oehmen *et al*., 2005; Lopez-Vazquez *et al*., 2009a). On the other hand, *Defluviicoccus*-related organisms have also been reported as dominant in bioreactors with acetate as the sole carbon source (Dai *et al*., 2007; Wang *et al*., 2008). As yet, there is no clear consensus on the competition between γ-GAOs and *Defluviicoccus*-related organisms for acetate. Perhaps, the outcome may depend on other environmental factors, like temperature, high COD/P ratios or different dissolved oxygen concentrations during the famine period as Oehmen and colleagues (2007) already suggested.

One problem in the analysis of the phylogeny of *Defluviicoccus*-related organisms is the recognition of separate subgroups. It is therefore unclear, whether the same subgroup was present in previous studies and if there are major functional differences between the subgroups. Moreover, in granular sludge, significant simultaneous denitrification occurs, suggesting that the microbial community inside the granules mainly experiences anaerobic/anoxic conditions, which have hardly been studied under controlled conditions.

An extra complication in the research on *Defluviicoccus*-related organisms is the difficulty to obtain its DNA using standard extraction methods (McIlroy *et al*., 2008). In this study, the DNA extraction of *Defluviicoccus*-related organisms (B24) from the granules with the standard extraction procedure also led to unsatisfactory results. Meyer and colleagues (2006) also reported DNA extraction problems, even from sludge that comprised more than 50% of *Defluviicoccus*-related organisms. To successfully identify *Defluviicoccus*-related organisms in cluster II, rRNA-based stable isotope probing (SIP) followed by full-cycle rRNA analysis was needed (Meyer *et al*., 2006). In the present study, the problem was solved by using liquid nitrogen in the extraction procedure, omitting arduous methods which include SIP, chloroform, phenol or sodium trichloroacetate (McMahon *et al*., 2002; McIlroy *et al*., 2008).

This seems to suggest that this particular cluster (II) within the *Defluviicoccus*-related organisms group is difficult to extract using normal extraction kits. This raises the question whether or not the cluster has been overlooked in community analysis studies in the past. Moreover, this shows again that genomic results should always be verified by complementary methods like FISH to ensure adequate interpretation of experimental results.

### What is the cause of the segregation?

Segregation of biomass in different types of granules in a laboratory-operated AGS system have recently also been reported by Barr and colleagues (2010) and Winkler and colleagues (2011). The segregation as reported by Winkler and colleagues (2011) was due to different microbial populations in fast and slow settling particles, largely due to differences in particle size and to lesser extent density variations. In this case, the slower settling microbial population was assumed to be exposed to different substrate concentrations during feeding, due to its localization in the top of the sludge bed. Furthermore, the fraction of cells that is exposed to oxygen during the aerobic period depends a lot on the particle size as well. Moreover, due to sludge removal during settling at a specific height in the reactor, both populations get a different SRT. In the case described in this study, as well as in the study by Barr and colleagues (2010), both types of granules were fully mixed and no difference in feeding regime or SRT between the different granules occurs. Additionally, Barr and colleagues (2010) noticed that white granules comprised more than 97% *Candidatus Accumulibacter phosphatis* (PAO) and about 0.9% of *Competibacter phosphatis* (γ-GAO), as measured by quantitative FISH, while in the yellow granules, these species were distributed vice versa, 12% and 58% respectively. Barr and colleagues (2010) suggested that the separation was partly induced by excessive production of exopolysaccharides (EPS) by PAOs and the segregation could not be attributed to specific operational reasons. Winkler and colleagues (2011) also indicated a segregation for the same microbial populations in top and bottom granules, induced by a difference in density of the granules and thereby a difference in settling velocity. In addition, PAOs seemed to proliferate in smooth white granules.

In our case, there is no big difference in settling velocity as inferred from the mixed presence of both granule types in the settling bed. We postulate therefore that the difference in EPS produced by the different species is the reason for this separation, as Barr and colleagues (2010) already suggested. Initially, white micro colonies grew on the surface of the black granules, but as the conditions changed, these micro-colonies gradually detached and became independent granules. Substantial amounts of EPS are visible around the *Defluviicoccus*-related clusters as illustrated in Fig. 6.

It could be hypothesized that *Defluviicoccus*-related organisms in cluster II are excreting EPS with different characteristics compared with that of for example the γ-GAOs, which were found mainly in the black granules. This could also lead to segregation if the two polysaccharides produced repulse each other. This idea is also supported by Seviour and colleagues (2012; 2011), who postulated that there might be several EPS produced by different bacteria that have variable properties. *Defluviicoccus*-related organisms themselves are known for their clustering and heavy encapsulation with EPS (McIlroy *et al*., 2008). Initial extraction of EPS as reported by Lin and colleagues (2010) before showed a large fraction of alginate like polymers (> 15%) in the black granules while the white granules had no or very limited alginate like polymer content (< 4%) (results not shown). Clearly, besides alginate-like EPS other polymers could be used by bacteria to construct granular sludge. Further experiments on the EPS and species present in the two different granule types should elucidate the segregation behaviour in the future.

## Experimental procedures

The experimental setup and analytical procedures are identical as described in Pronk and colleagues (2014). In short, the sequencing batch reactor (2.7 l) cycle consisted of a 60 min feeding, 112 min aeration, 3 min settling and a 5 min effluent discharge period. The pH was controlled at 7 by addition of 1 M HCl and 1 M NaOH. The dissolved oxygen concentration was controlled at 50% saturation. The temperature of the reactor was controlled at 35- ± 1°C. An average SRT of 24 days was maintained by manually removing biomass from the reactor on a weekly basis. The SRT was calculated by the total biomass present in the system, removed via the effluent and the biomass that was manually removed. The reactor was inoculated with granular sludge from a previous experiment on a complex carbon medium, which contained acetate, methanol, propanol, butanol, propionaldehyde and valeraldehyde (Pronk *et al*., 2015). Granule size distribution was performed by measuring at least 500 granules in a mixed reactor sample using a Leica Microsystems stereo zoom microscope (M205 FA) in combination with Leica Microsystems Qwin (V3.5.1) image analysis software.

### Detection of methanogenic activity in batch

Granules from the reactor were separated manually and transferred to reactor medium without a carbon source in a 20 ml glass bottle. The pH was set to 7.2. The bottles were then flushed with nitrogen gas to make them anaerobic. Methanol (20 mM) was injected and the bottles were incubated in a shaker set at 190 r.p.m. at 35°C. Samples were taken from the headspace at regular time intervals and the methane concentration in the headspace was determined on a Varian 3800 gas chromatograph. Gas samples were injected with a 100 μl gastight Hamilton syringe in a Varian Ultimetal 1079 split/splitless, which was operated at 200°C at a split ratio of 100. A CPSil5CB 5 (50 m × 0.32 mm) capillary column was used isothermally at 100°C at a constant gas flow rate of 10 ml min^−1^. The used carrier gas was helium. Methane peaks were detected with a Varian flame ionisation detector that was operated at 300°C. The helium make-up flow was 25 ml min^−1^, hydrogen flow was 30 ml min^−1^ and air flow was 300 ml min^−1^.

### Medium

The synthetic medium consisted of 150 ml medium A and 150 ml medium B dosed (Table 1) together with 1200 ml heated tap water, achieving an influent temperature of 35°C. Influent was fed to the bottom of the reactor column. No additional mixing was applied during this period. The carbon composition of medium A consisted of a mixture of methanol and acetate (Table 1). Trace elements solution had the following composition: 63.7 g l^−1^ C_10_H_14_N_2_Na_2_O_8_●2H_2_O (EDTA TITRIPLEX® III) (171.1 mM), 4.99 g l^−1^ FeSO_4_.7H_2_O (17.95 mM), 2.2 g l^−1^ ZnSO_4_.7H_2_O (7.6 mM), 7.34 g l^−1^ CaCl_2._2H_2_O (49.9 mM), 5.06 g l^−1^ MnCL_2_.4H_2_O (51.1 mM), 1.51 g l^−1^ Na_2_MoO_4_.2H_2_O (6.2 mM), 1.57 g l^−1^ CuSO_4_.5H_2_O (6.3 mM), 3.22 g l^−1^ CoCl.6H_2_O (15.9 mM).

### Fluorescent *in situ* hybridization

The FISH probes used in this study to show all present bacteria is a mixture of EUB338, EUB338II and EUB338III, referred to in the text as EUBmix. DF1mix (*Defluviicoccu*s-related organisms in cluster I) consisted out of TFO_DF218 plus TFO_DF618 (Wong *et al*., 2004) and DF2mix (*Defluviicoccu*s-related organisms in cluster II) consisted out of DF988, DF1020, helper probes H966 and H1038 (Meyer *et al*., 2006) to target tetrad-forming *α-Proteobacteria*. GAO belonging to *γ-Proteobacteria* were stained using GAO-Q431 and GAOQ-989 (in text as GAOmix) probes developed by Crocetti and colleagues (2002). GAM42A and BET42A developed by Manz and colleagues (1992) were used to detect *γ-Proteobacteria* and *β-Proteobacteria* in the samples. All probes were prepared with a concentration of 35% formamide. Due to difficulty in the adhesion of the biomass to the glass slides, the drying time was done overnight at 45°C. The samples were examined with a Zeiss Axioplan 2 epifluorescence microscope equipped with filter set 26 (bp 575–625/FT645/bp 660–710), 20 (bp 546/12/FT560/bp 575–640), 17 (bp 485/20/FT 510/bp 5515–565) for Cy5, Cy3 and fluos respectively.

### Sampling, nucleic acids extraction and PCR amplification

Granules taken from the reactor were separated (black and white) and pottered to create a cell suspension. The cell suspension was washed two times with Phosphate Buffered Saline (PBS) buffer (pH 7). The supernatant was discarded and the pellet was stored at −80°C. Default (routine) DNA extractions were performed using the Ultraclean Microbial DNA extraction kit from Mobio (USA) according to manufacturer’s recommendations.

### Modified DNA extraction method using liquid nitrogen

A more stringent extraction was also performed by modifying the standard extraction protocol. The pellet was incubated with 3 mg ml^−1^ Lysozyme for 30 min at 37°C at 800 r.p.m. (Sigma Aldrich L7651) in a bead buffer followed by Proteinase-K (500 mg ml^−1^) digestion and 0.5% Sodium Dodecyl Sulphonate (SDS) incubation of 15 min at 56°C at 800 r.p.m. (#EO0491, Thermo scientific). The subsequent, lysate was transferred into resistant metal bead tubes with a special silicon cap (Biospec, Cat. No. 2007/2008) and frozen into liquid nitrogen with small beads supplied with the kit, [∼ 50 mg large volcanic beads from the Ultraclean soil DNA extraction kit and 2 × 3.2 mm chrome-steel beads (Cat. No. 11079132c)]. Before bead beating the resulting 50 μl solution, MD1 was added. The total sample was homogenized on a Mini-Beadbeater-16 (Biospec, USA) for 5 min and refrozen with liquid nitrogen and the procedure was repeated another time. After the second homogenizing step, the sample was refrozen and homogenized for a final 3 min. The resulting lysate was extracted using the Ultraclean Microbial DNA extraction kit (Mobio, USA) as mentioned above by following the supplied protocol from step 6 on.

After extraction 5 μl of a total of 50 μl was subjected to gel electrophoresis to check for quality and quantity. PCR amplification of the 16s rDNA gene from the whole bacteria kingdom was performed as described in Bassin and colleagues (2011). Sequences were deposited into GenBank under Accession No. KP064478 – KP064500.

## Conclusions

Stable AGS formation was shown for mesophilic conditions. Controlling the anaerobic SRT in an AGS reactor below 10 days (at 35°C) enabled prevention of growth of methylotrophic methanogens, thereby preventing methane emissions. No detectible anaerobic methanol conversion occurred in the absence of methanogens. Aerobic oxidation of methanol did not deteriorate the stability of the sludge granules. *Defluviicoccus*-cluster II-related organisms belonging to the *α-Proteobacteria* replaced the *γ-Proteobacteria* (so called *γ*-GAOs) when the SRT was decreased. Remarkably, the two GAO types of organisms grew segregated in two clearly distinct types of granules. In addition, we demonstrated that a liquid nitrogen extraction step proved an easy and reliable method to obtain DNA from *Defluviicoccus*-related organisms in cluster II, since the standard extraction procedure led to unsatisfactory results. This work further highlights the potential of AGS systems to effectively influence the microbial communities through sludge age control in order to optimize the wastewater treatment processes.

## Conflict of Interest

The authors do not have any conflict of interest related to the work presented in this paper.

## Supporting Information

**Fig. S1.** Cycle measurement with anaerobic conversion of methanol to methane. **Fig. S2.** Cycle measurement with aerobic conversion of methanol.
