# Supplementary material for: Effect of sludge age on methanogenic and glycogen accumulating organisms in an aerobic granular sludge process fed with methanol and acetate
Source: Microb Biotechnol. 2015 Jun 8;8(5):853–64. doi: 10.1111/1751-7915.12292 (PMC4554473; doi:10.1111/1751-7915.12292)
Supplement: Supplementary file 1 [file mbt20008-0853-sd1.docx]

**PREVENTING METHANOGENESIS BY SRT CONTROL AND ITS EFFECT ON THE MICROBIAL COMMUNITY IN AN AEROBIC GRANULAR SLUDGE REACTOR FED WITH ACETATE AND METHANOL**

M. Pronk,^1^* B. Abbas^1^, R. Kleerebezem^1^, M.C.M van Loosdrecht ^1^*

Department of Biotechnology, Delft University of Technology, Delft, The Netherlands^1^ and Royal HaskoningDHV B.V., P.O Box 1132, 3800 BC Amersfoort, The Netherlands^2^

* Corresponding author. Mailing address: Delft University of Technology, Department of Biotechnology, Julianalaan 67, Delft, 2628 BC, The Netherlands. Phone: 31152781618. Fax: 31152782355. E-mail: [M.pronk@tudelft.nl](mailto:M.pronk@tudelft.nl), [M.C.M.vanLoosdrecht@tudelft.nl](mailto:M.C.M.vanLoosdrecht@tudelft.nl)

Figure S1 Cycle measurement with anaerobic conversion of methanol to methane


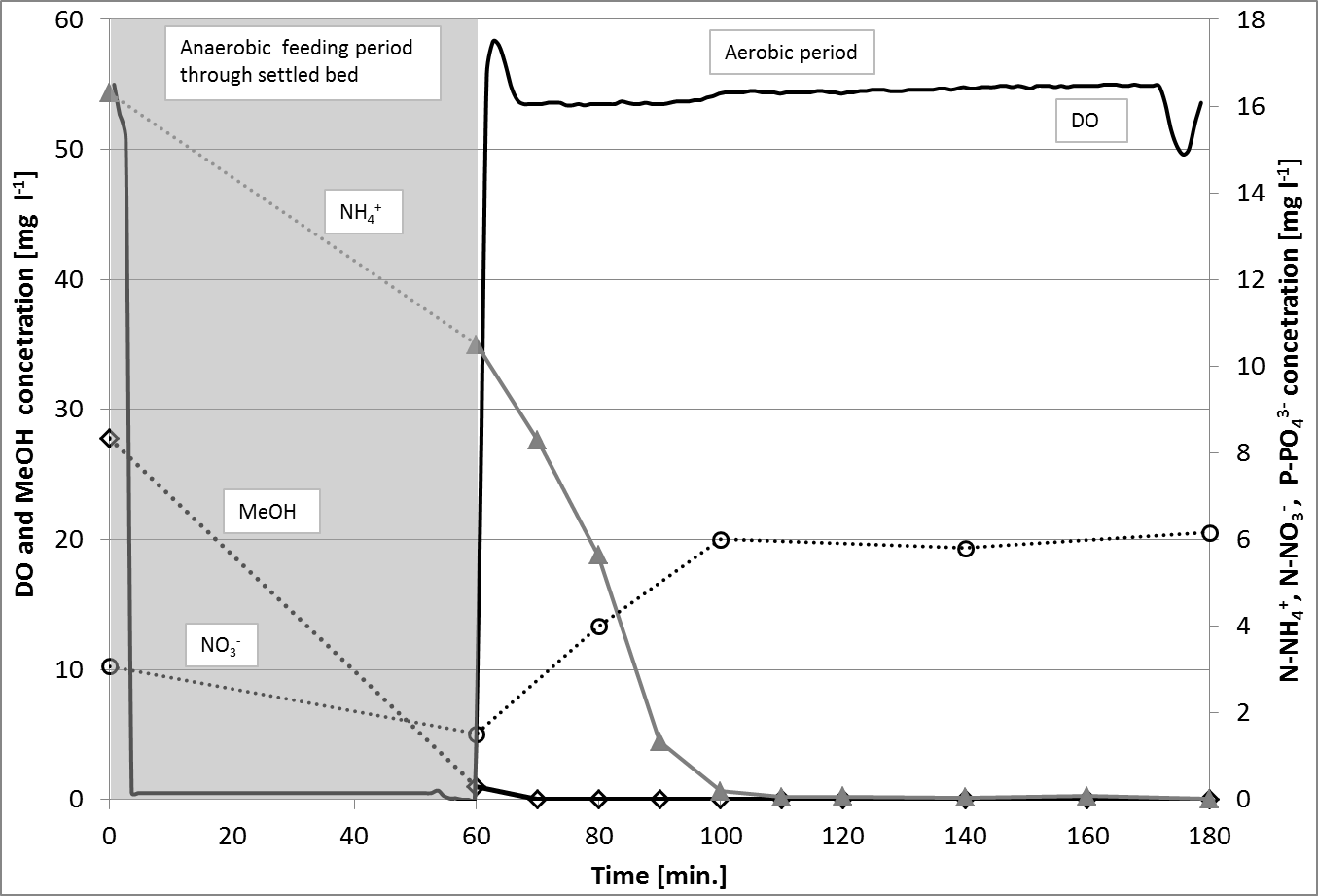


The starting ammonium, nitrate and methanol concentrations depicted at time zero were calculated based on the concentration in the inﬂuent, effluent and the dilution in the reactor after the start of the aeration.

Figure S2 Cycle measurement with aerobic conversion of methanol


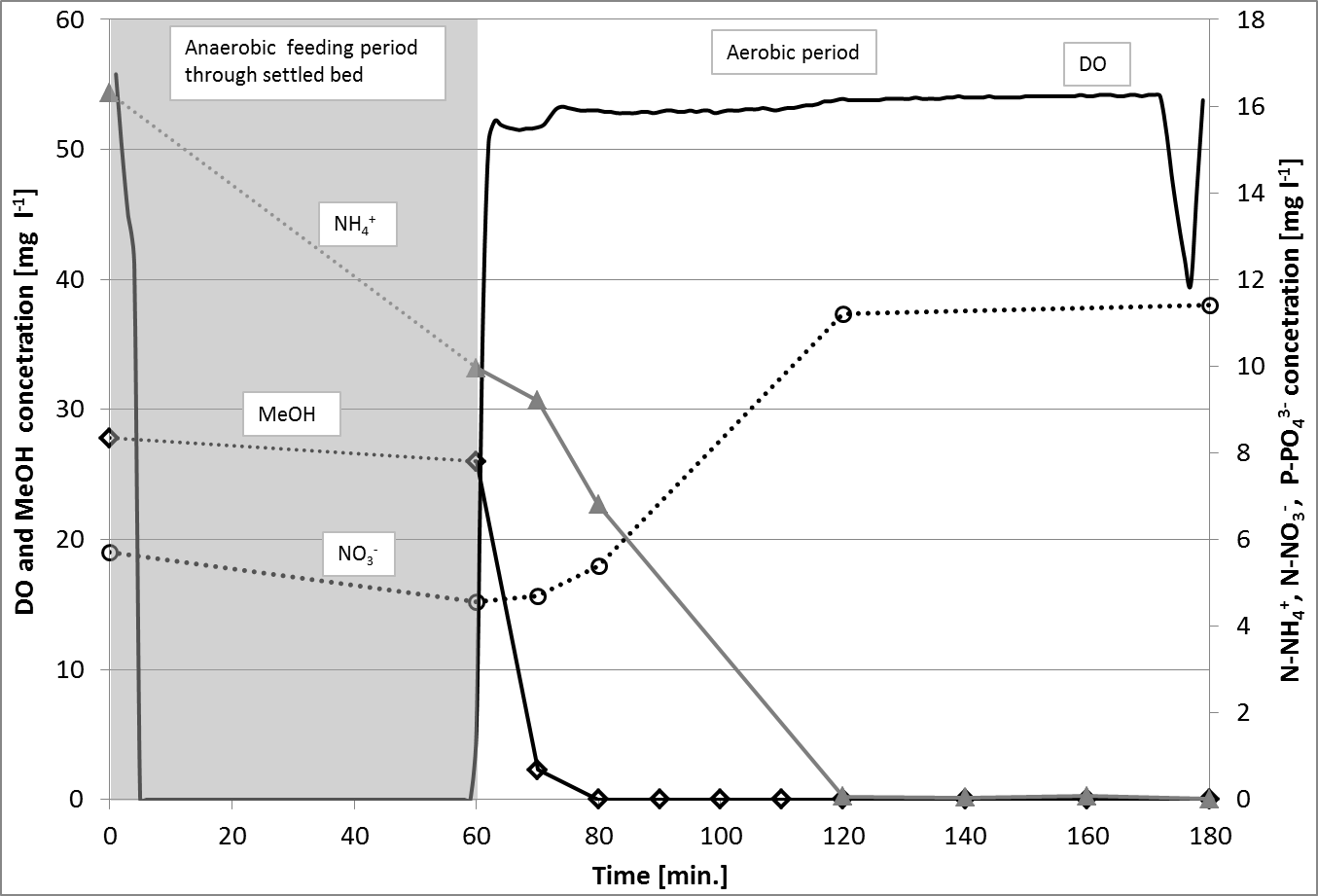


The starting ammonium, nitrate and methanol concentrations depicted at time zero were calculated based on the concentration in the inﬂuent, effluent and the dilution in the reactor after the start of the aeration.
